# Genetics and Molecular Pathogenesis of the Chondrosarcoma: A Review of the Literature

**DOI:** 10.3390/cimb46110751

**Published:** 2024-11-08

**Authors:** Georgian-Longin Iacobescu, Antonio-Daniel Corlatescu, Bogdan Serban, Razvan Spiridonica, Horia Petre Costin, Catalin Cirstoiu

**Affiliations:** 1 Department of Orthopedics and Traumatology, Carol Davila University of Medicine and Pharmacy, 050474 Bucharest, Romania; georgianyak@yahoo.com (G.-L.I.); antonio.corlatescu@gmail.com (A.-D.C.); rspiridonica@yahoo.com (R.S.); horia-petre.costin0720@stud.umfcd.ro (H.P.C.); catalin.cirstoiu@umfcd.ro (C.C.); 2 University Emergency Hospital, 050098 Bucharest, Romania

**Keywords:** chondrosarcoma, biomarkers, genetic alterations, molecular pathways, personalized therapy

## Abstract

The chondrosarcoma, a cartilage-forming bone tumor, presents significant clinical challenges due to its resistance to chemotherapy and radiotherapy. Surgical excision remains the primary treatment, but high-grade chondrosarcomas are prone to recurrence and metastasis, necessitating the identification of reliable biomarkers for diagnosis and prognosis. This review explores the genetic alterations and molecular pathways involved in chondrosarcoma pathogenesis. These markers show promise in distinguishing between benign enchondromas and malignant chondrosarcomas, assessing tumor aggressiveness, and guiding treatment. While these advancements offer hope for more personalized and targeted therapeutic strategies, further clinical validation of these biomarkers is essential to improve prognostic accuracy and patient outcomes in chondrosarcoma management.

## 1. Introduction

Chondrosarcomas are malignant neoplasms of cartilage origin characterized by diverse morphological features and variable clinical behavior. They represent approximately 20% of all primary bone malignancies [1], typically arising in the pelvis or long bones [2]. Chondrosarcomas are classified as either primary (conventional) or secondary tumors. Primary chondrosarcomas develop in previously normal bone, while secondary chondrosarcomas arise from preexisting lesions, such as enchondromas or osteochondromas [1]. Conventional chondrosarcomas, comprising 85–90% of cases, are categorized into central, periosteal, and peripheral subtypes [3]. Non-conventional variants include clear cell, mesenchymal, and dedifferentiated chondrosarcomas [3]. Radiographic imaging often reveals characteristic features of chondrosarcomas, allowing for a definitive diagnosis based on imaging alone.

Conventional chondrosarcoma (CHS) of the bone is the most common type of primary CHS, typically affecting an older population, with most patients over 50 years old. The peak incidence occurs between the fifth and seventh decades of life, and there is a notable male predilection, with a ratio of 1.5–2:1. CHS can involve any bone, although the incidence of axial and appendicular involvement is similar, with the pelvis, especially the ilium, frequently affected. Long tubular bones, including the proximal femur, the proximal humerus, and the distal femur, are also commonly involved. In contrast, CHS in smaller tubular bones, such as those in the forearm, clavicle, and sesamoids, is extremely rare (1–4% of all cases) [4].

The histological grading of CHS is primarily based on nuclear size, hyperchromasia, cellularity, and mitoses. Low-grade (grade 1) lesions are poorly cellular with small, hyperchromatic nuclei, and they lack mitotic figures. Intermediate (grade 2) tumors are more cellular, with nuclear enlargement and rare mitotic activity, while high-grade (grade 3) tumors show increased mitotic figures and necrosis. Genetic aberrations become more prevalent as CHS progresses from low- to high-grade, with p53 mutations and loss of INK4A/p16 expression commonly associated with high-grade CHS. For prognosis, the histological grade is the most important predictor of recurrence and metastasis, with higher-grade tumors significantly linked to a higher probability of metastasis [4].

Efforts to identify reliable molecular markers and therapeutic targets for CHS have explored collagen subtypes, with types II and X, along with aggrecan, proposed as markers of a mature neoplastic phenotype, while collagen type I is associated with a proliferative, dedifferentiated state [5]. Cyclooxygenase-2 overexpression has been linked to a higher histologic grade and poorer survival but lacks independent prognostic value [6]. Attempts to inhibit CHS growth using celecoxib were unsuccessful [7], although the Hedgehog signaling pathway remains a potential therapeutic target [8]. Activation of the IHH/PTHLH pathway and bcl2 reactivation are implicated in CHS progression, with bcl2 serving as a marker to differentiate low-grade CHS from enchondromas [9]. As CHS progresses from low- to high-grade, genetic aberrations increase, with p53 overexpression and TP53 mutations occurring late in high-grade cases [8,10,11]. Aberrations, such as 12q13 amplification (involving MDM2) and 9p21 loss (affecting CDKN2A/p16/INK4A), are common, with loss of p16 linked to high-grade CHS [12]. Prognosis is primarily determined based on the histological grade, with grade 1 tumors having an indolent course and no metastatic risk, while grades 2 and 3 are associated with metastasis and lower survival rates [13]. Surgical excision remains the mainstay of treatment, with wide excision for high-grade CHS and curettage for grade 1 cases yielding effective long-term control [14].

The tumor microenvironment (TME) in chondrosarcomas is characterized by a dense and heterogeneous extracellular matrix (ECM) that supports tumor growth and metastasis through various signaling pathways. Unlike normal chondrocytes, chondrosarcoma cells create a compact matrix rich in type II collagen, hydrophobic proteoglycans, and hyaluronan, contributing to the tumor’s structural integrity and invasive potential. ECM remodeling is regulated by matrix metalloproteinases (MMPs), with high levels of MMP-1, MMP-2, and MMP-13 correlating with increased tumor cell invasiveness, while MMP-9 expression has been linked to better survival outcomes. Additionally, cytokines like IL-1β and chemokines like CCL-5 drive tumor vascularization and growth by stimulating vascular endothelial growth factor (VEGF) release [15,16]. Hypoxia, common in the chondrosarcoma TME, activates hypoxia-inducible factors (HIF-1α and HIF-2α), which further promote metastasis by triggering pathways involved in cancer cell dormancy, angiogenesis, and ECM remodeling, as well as contributing to chemoresistance through cancer stem cell survival [17].

The immune microenvironment in chondrosarcomas also plays a crucial role in tumor progression, with tumor-associated macrophages (TAMs) representing the predominant immune cell type. Most TAMs exhibit a pro-tumoral M2-like phenotype, supporting angiogenesis, immune suppression, and tumor cell proliferation while suppressing CD8+ cytotoxic T-cell activity through cytokines and reactive metabolites. Additionally, immune cells, such as T-cells and natural killer cells, are often located at the tumor’s periphery, possibly due to the dense ECM barrier. However, dedifferentiated chondrosarcomas, with less organized ECM structures, may show immune cells interspersed with tumor cells, suggesting potential responsiveness to immunotherapy in some cases. Recent studies have highlighted markers like CSF1R in TAMs as potential targets for immunomodulation. At the same time, the presence of regulatory T-cells (Tregs) and immunosuppressive factors like PD-L1 further suggests that immune checkpoint inhibitors could provide a therapeutic benefit by reversing immune evasion in chondrosarcomas [18,19].

## 2. Genetic Alterations in Chondrosarcomas

### 2.1. IDH1/IDH2 Mutations

In recent years, recurrent heterozygous hotspot mutations in isocitrate dehydrogenase 1 and 2 (IDH1 and IDH2) genes, specifically at residues p.R132 and p.R140/p.R172, respectively, have been frequently identified in cartilage tumors, such as enchondromas, central conventional chondrosarcomas, and dedifferentiated chondrosarcomas. These mutations are found in 87% of enchondromas, around 50% of central conventional chondrosarcomas, and over 80% of dedifferentiated chondrosarcomas [20]. The early occurrence of IDH mutations suggests that they play a pivotal role in tumorigenesis by promoting chondrogenic differentiation while inhibiting osteogenic pathways in mesenchymal stem cells, the likely progenitors of these tumors [21,22]. Despite their significance in the early stages of tumor formation, the prognostic implications of IDH mutations in chondrosarcomas remain controversial. Some studies suggest that IDH mutations do not affect outcomes [23], while others report either worse [24] or better [25] prognoses in patients with IDH-mutant chondrosarcomas. This variability could be due to differences in chondrosarcoma subtypes, follow-up periods, and detection techniques, such as Sanger sequencing, which might not capture mutations in samples with low variant allele frequency.

IDH1 and IDH2 mutations play a significant role in the progression and molecular characteristics of chondrosarcomas, as identified in recent multi-omics studies. These mutations, often found in cartilage tumors, particularly in central chondrosarcomas, lead to widespread hypermethylation across the genome. This hypermethylation affects key cellular processes, including differentiation and proliferation. Interestingly, different IDH mutations are associated with varying tumor aggressiveness, with IDH2 mutations (R172S/W/T) notably linked to more advanced and dedifferentiated forms of chondrosarcoma. Despite these molecular insights, the presence of IDH mutations does not directly correlate with a worse overall prognosis in all cases. However, tumors with dedifferentiated histology, which often display these mutations, tend to have a poorer outcome, indicating that IDH mutations contribute to more aggressive tumor phenotypes [26].

IDH mutations are identified in 52–59% of central chondrosarcomas (CSs) and 57% of dedifferentiated CSs [27]. The occurrence of IDH mutations in both benign enchondromas and malignant CSs suggests that these mutations are an early event, indicating that cartilaginous neoplasms may exist on a spectrum of malignant potential. Additionally, IDH mutations are also observed in gliomas, acute myeloid leukemia (AML), and cholangiocarcinomas [28]. IDH, an enzyme in tricarboxylic acid (Krebs cycle), normally converts isocitrate to α-ketoglutarate (α-KG). However, mutant IDH (mIDH) loses the ability to perform this conversion and acquires a new function that results in the accumulation of δ-2-hydroxyglutarate (D2HG). D2HG is considered an oncometabolite because it inhibits α-KG-dependent dioxygenases, which are critical for DNA and histone demethylation, leading to a hypermethylated state in both DNA and histones [29]. Nevertheless, IDH mutations alone are insufficient for malignant transformation, similarly to the loss of EXT. Targeting oncogenic mIDH1/2 offers a potential therapeutic approach, with several inhibitors currently being tested in clinical trials for patients with AML or solid tumors, including CS [30].

### 2.2. COL21A

The *COL2A1* gene, which is responsible for encoding the alpha-1 chain of type II collagen, plays a pivotal role in developing chondrosarcomas (ChSs). Mutations in *COL2A1* are commonly observed in ChS, especially in higher-grade tumors, where they cause significant disruptions in the collagen structure and the extracellular matrix (ECM). These alterations in ECM deposition and signaling can drive tumor progression by impairing normal cartilage differentiation processes, potentially facilitating oncogenesis. Notably, these mutations are associated with more aggressive and malignant tumor behavior. However, despite their biological importance, *COL2A1* mutations have yet to be adopted as clinical biomarkers for prognosis prediction [26].

The second most common mutations in chondrosarcomas (ChSs) are found in the *COL2A1* gene, which encodes the alpha-1 chain of type II collagen fibers in cartilage. These mutations have been identified in 37% of ChS cases [31,32]. Studies have revealed truncating, essential splice-site, and missense mutations in high-grade conventional ChS and dedifferentiated ChS. These mutations lead to significant disruption in extracellular matrix (ECM) deposition and signaling, contributing to oncogenesis by interfering with normal differentiation processes in cartilage tissue [31]. Furthermore, an analysis of a single *COL2A1* gene showed a tumor mutational burden (TMB) ranging from 1 to 115 mutations per case. Among the identified somatic mutations in this gene are missense, nonsense, splice-site, and synonymous mutations, as well as indels and substitutions in microRNAs. Notably, somatic mutations doubled in dedifferentiated ChS and grades 2 and 3 ChS compared to grade 1 ChS. Despite these findings, further detailed research is still required [31].

### 2.3. TP53 and CDKN2A

In chondrosarcomas (ChSs), mutations in the *TP53* gene are observed in approximately 22% of cases, particularly in higher-grade tumors, such as grade 2/3 and dedifferentiated chondrosarcomas. The *TP53* gene is a tumor suppressor that maintains genomic stability by regulating cell cycle progression and apoptosis. Mutations in *TP53* often lead to the loss of its tumor-suppressing function, contributing to the malignant progression of ChS. These alterations are typically absent in well-differentiated tumors, indicating that *TP53* mutations are associated with more aggressive forms of the disease. Despite the clear role of *TP53* in tumor progression, its mutations have limited value in survival prediction or risk stratification [32].

Gene aberrations are common in chondrosarcomas (ChSs), with higher grades showing more abnormalities [33]. Loss of heterozygosity in the 17p1 region, leading to *TP53* loss, occurs in about 25–30% of high-grade ChS [34,35], along with mutations in TP53 introns and exons [36]. High-grade ChS also often amplifies in the 12q13 region involving MDM2 and deletions in the 9p21 region containing *CDKN2A* [33]. Loss of *CDKN2A*/p16INK4A is crucial for ChS progression [37], and p16INK4a hypermethylation is frequently observed in ChS.

Alterations in *CDKN2A* and *TP53* are common in high-grade ChS but have limited prognostic value [31,38]. Near-haploid karyotypes have been reported, although their significance remains unclear [39,40].

In contrast to chondrosarcomas, osteosarcomas exhibit a higher degree of genomic instability, marked by frequent chromosomal rearrangements and elevated mutation rates in key cell cycle regulatory genes, such as *TP53*, *PTEN*, and *CDKN2A*/B. Disruptions in the *TP53* pathway, often through mutations and deletions, lead to uncontrolled cell proliferation. Another defining feature of osteosarcomas is the WNT/β-catenin signaling pathway, where mutations contribute to abnormal cell differentiation and accelerated tumor growth. The IGF1/IGF2 and IGF1R pathways also play crucial roles, with upregulation fostering aggressive proliferation and tumor survival. Significant differential expression of genes like *BTNL9*, *MMP14*, *ABCA10*, *ACACB*, *COL11A1*, and *PKM2* further highlights osteosarcomas’ unique molecular profile, positioning these genes as potential biomarkers. Together with targets in the IGF1R and WNT pathways and TP53-related regulators, these features reveal promising therapeutic strategies distinct from those used for chondrosarcomas [41,42,43].

## 3. Pathway Dysregulation in Chondrosarcomas

Large-scale transcriptomic and proteomic analyses, including whole-exome sequencing and immunohistochemistry, have identified frequent aberrations in tumor suppressors and cell cycle regulators in ChS cells [44,45]. Key genes, including *CDK4* and *MDM2*, are implicated in the pRb and p53 pathways [44]. The degradation of p53 via *MDM2* has been linked to tumor progression in central ChSs, and p53 deficiency can lead to ChS arising from benign lesions like enchondroma [46]. With *MDM2* upregulated in one-third of high-grade ChSs and correlating with more aggressive disease, inhibitors targeting the p53–*MDM2* interaction (e.g., RG7112) could be a promising therapy [47].

The pRb pathway, disrupted in many cases of high-grade ChS, involves loss of *RB1* gene heterozygosity, reduced *CDKN2A*/p16 expression, or increased *CDK4* or cyclin D1 expression [44,45]. *CDK4/6* inhibitors (palbociclib, ribociclib, abemaciclib), already approved for metastatic breast cancer, are considered potential treatments for advanced ChS [48].

The SRC signaling cascade, crucial in sarcoma survival, migration, and proliferation, regulates PI3K–AKT signaling. Dasatinib (BMS-354825), a tyrosine kinase inhibitor targeting SRC and ABL, has shown efficacy in reducing ChS cell viability, although it did not induce caspase-3-mediated apoptosis [49,50]. Dasatinib also sensitized mutant p53 ChS cells to doxorubicin, inhibiting migration and inducing apoptosis in vitro [50]. However, despite the potential of SRC pathway inhibition, a phase II trial of dasatinib in high-grade sarcoma patients showed no clinical benefit [51].

The Hedgehog (HH) pathway is essential for embryogenesis and adult tissue maintenance. In mammals, Indian Hedgehog (IHH) binds to Patched (PTCH1), activating Smoothened (SMO). This triggers GLI transcription factors, leading to various cellular responses, like survival and differentiation [52].

Chondrogenesis is regulated by the IHH/PTHrP pathway, where IHH promotes chondrocyte division and PTHrP inhibits differentiation in a feedback loop. Given its role in chondrogenesis, targeting HH signaling in chondrosarcomas (CSs) has been explored. SMO inhibitors like HPI-4 and IPI-926 showed efficacy in preclinical models, but clinical trials were unsuccessful [53,54,55]. Despite setbacks, more potent inhibitors are in development [52].

RTK activation triggers PI3K, leading to Akt activation, which regulates survival and growth. PTEN mutations are rare in CS, but active Akt signaling is present. mTOR kinase, which integrates PI3K/Akt signals, activated 69% of conventional and 44% of dedifferentiated CS samples [56]. The dual PI3K/mTOR inhibitor BEZ235 induced G1 arrest in CS cell lines without causing apoptosis [56]. Clinically, sirolimus combined with low-dose cyclophosphamide stabilized disease in 60% of patients with advanced CS for at least 6 months [57]. Further research into PI3K–Akt–mTOR pathway inhibition in CS is needed.

## 4. Epigenetic Changes in Chondrosarcomas

DNA methylation, the addition of a methyl group to DNA catalyzed by DNA methyltransferases (DNMTs), plays a significant role in gene regulation [58,59]. Hypomethylation, the loss of this methyl group, can occur globally or in specific genes and is involved in tumor progression, as seen in repetitive DNA sequences like Satellite 1 and L1 [60,61]. DNMT inhibitors, such as azacytidine and decitabine (DAC), are FDA-approved for treating hematological conditions like acute myeloid leukemia (AML) and myelodysplastic syndrome (MDS) [62]. In chondrosarcomas (CSs), DAC treatment has been shown to hypomethylate Satellite 1 and L1 and overexpress genes like Sox-2 and midkine (MDK), leading to increased tumor invasiveness in some models while inhibiting growth in others [63,64]. DAC have also been associated with decreased methylation at CpG sites, leading to increased expression of epithelial markers like Maspin and 14-3-3σ, proteins involved in cell adhesion, apoptosis, and cell cycle control, suggesting a role in mesenchymal to epithelial transition (MET) [65]. Additionally, the tumor microenvironment can influence methylation patterns, as studies in SRC cells transplanted into different sites showed variations in gene expression and hypomethylation compared with normal cartilage [66]. While DAC show promise in some contexts, their role in promoting or inhibiting CS progression remains complex and warrants further research [66].

DNA methylation, adding a methyl group to CpG sites in DNA, regulates gene expression and plays a significant role in tumorigenesis, including in cancers like lung, breast, liver, and colon cancer, as well as melanoma and glioma [67]. Hypermethylation, in particular, is associated with mutations in genes like IDH1 and IDH2, which are frequently altered in cancers, such as acute myeloid leukemia, glioma, and chondrosarcomas (CSs) [68]. Mutant IDH leads to the production of the oncometabolite D2HG, which inhibits α-KG-dependent enzymes, causing DNA hypermethylation and histone modification, ultimately contributing to malignancy [69]. Studies have linked IDH mutations in CS to hypermethylation of genes involved in stem cell maintenance and differentiation, with potential therapeutic targets identified, including TET enzymes, Aurora kinase, and HDAC inhibitors [70]. Furthermore, hypermethylation of key genes like p16INK4, E-cadherin, and NAMPT has been observed in CS, suggesting potential for NAMPT and NAPRT inhibitors in high-grade CS treatment [71]. RUNX3, a tumor suppressor gene, is often silenced through hypermethylation in CS, and restoring its expression has been shown to inhibit proliferation and induce apoptosis [72]. Additionally, hypermethylation of p73, a member of the p53 tumor suppressor family, correlates with CS progression, making it a potential prognostic marker and therapeutic target [73].

MicroRNAs (miRNAs) are small, noncoding RNAs (18–24 nucleotides) that regulate gene expression by binding to the 3′ untranslated region (3′ UTR) of target mRNAs. Each miRNA can target multiple mRNAs, with around 600 miRNAs regulating about 60% of human genes. miRNAs are involved in normal chondrogenesis, such as miR-140, which regulates HDAC4 to promote chondrocyte hypertrophy via RUNX2. In chondrosarcomas (CSs), miRNAs also influence oncogenesis. For example, miR-100 suppresses tumors by targeting mTOR but is downregulated in CS, while miR-30a inhibits tumor growth by targeting SOX4. miR-181a, an oncogene, promotes VEGF expression, aiding CS progression [32] (Table 1).

## 5. Targeted Therapies in Chondrosarcomas

Chemotherapy is generally ineffective for chondrosarcomas, and no standard systemic treatment exists for conventional cases due to factors like slow proliferation, multidrug resistance, high expression of anti-apoptotic proteins, and poor vascularity [74]. Clinical studies are challenging due to the rarity of the disease. Still, a retrospective study of nivolumab, with or without pazopanib, showed partial responses in 13% of sarcoma patients, including one with a dedifferentiated chondrosarcoma [75]. IL-8, a chemokine involved in tumor progression, was targeted in a phase 1 trial using BMS-986253, yielding a high disease control rate but no objective response, indicating a need for further research in chondrosarcoma patients [76].

Recent studies have identified potential therapeutic targets in chondrosarcomas. PPARγ activation has been shown to inhibit cell proliferation and induce apoptosis, with zaltoprofen demonstrating efficacy in activating PPARγ in chondrosarcoma cells [77,78]. CDK4 is highly expressed in chondrosarcomas and associated with poor prognosis. The CDK4 inhibitor palbociclib has shown promising results in halting cell cycle progression and inhibiting tumor cell proliferation and invasion [79].

Other promising treatments include MLN4924, a NEDD8-activating enzyme inhibitor, which has shown antitumor effects in both cell lines and a xenograft mouse model [80], and resveratrol, which inhibited cell viability and suppressed the STAT3 signaling pathway in chondrosarcoma cells [81]. The mTOR inhibitors rapamycin and everolimus have also demonstrated potential by reducing cell viability and suppressing tumor progression in both cell lines and animal models [82,83].

Despite limited advances in chondrosarcoma treatment, recent research has identified promising molecular targets for therapy. Activating peroxisome proliferator-activated receptor gamma (PPARγ) in chondrosarcoma cells—either directly or with drugs like zaltoprofen—has been shown to inhibit cell proliferation, induce apoptosis, and reduce invasion, marking it as a viable therapeutic pathway. Similarly, CDK4, linked to cell cycle regulation and associated with a poor prognosis in chondrosarcoma, can be targeted by palbociclib or CDK4-specific siRNA, which significantly reduces tumor cell proliferation and spread via cell cycle arrest. Inhibiting NEDD8, a ubiquitin-like protein, with MLN4924 also shows antitumor promise by promoting apoptosis and blocking tumor growth in preclinical models. The phytochemical resveratrol has also demonstrated tumor-suppressive effects by reducing cell viability and inhibiting STAT3 signaling. Targeting the mTOR pathway with inhibitors like rapamycin and everolimus further reduces tumor metabolism and progression, underscoring their potential to enhance chondrosarcoma treatment outcomes [84].

Immunotherapy in chondrosarcomas is emerging as a promising avenue of treatment, particularly for aggressive and metastatic forms of the disease, which are often resistant to conventional therapies, such as chemotherapy and radiation. Chondrosarcomas (CSs) exhibit several features, such as poor vascularization and the presence of multidrug-resistant pumps, that contribute to this resistance. However, immune checkpoint inhibitors (ICIs) like pembrolizumab and nivolumab have shown potential in clinical trials by targeting proteins, such as PD-1 and PD-L1, which are involved in tumor immune evasion. Early studies suggest that while response rates to immunotherapy in chondrosarcomas are still low, some patients with high-grade or dedifferentiated tumors have shown significant tumor regression [85] (Table 2).

Additionally, CDK4, a protein linked to cell cycle regulation, is highly expressed in chondrosarcomas and associated with metastasis and poor prognosis. Targeting CDK4 with palbociclib or CDK4-specific siRNA has demonstrated substantial reductions in chondrosarcoma cell proliferation, migration, and invasion by inducing cell cycle arrest in the G1 phase, mainly through the CDK4/Rb signaling axis. Furthermore, inhibiting the ubiquitin-like protein NEDD8 with MLN4924 has shown promising antitumor effects by reducing cell proliferation and enhancing apoptosis, even inhibiting tumor growth in xenograft models [79]. Other therapeutic targets include resveratrol, a phytochemical known for its antitumor effects across various cancers. In chondrosarcomas, resveratrol has been observed to decrease cell viability and proliferation, promote apoptosis, and inhibit STAT3 signaling, contributing to tumor suppression. The mTOR pathway, critical in cell growth and survival, has also been identified as a therapeutic target, with inhibitors like rapamycin and everolimus reducing cell metabolism, Glut1 and HIF1α expression, and overall tumor progression. These inhibitors have shown effectiveness in preclinical chondrosarcoma models, underscoring the mTOR pathway’s role in potentially improving treatment outcomes for this challenging malignancy [81].

## 6. Prognostic Biomarkers and Molecular Diagnostics

Surgery is the preferred treatment for chondrosarcomas, as these tumors are typically resistant to chemotherapy and radiation therapy. Patients with high-grade chondrosarcomas frequently experience local recurrence and distant metastasis following surgical resection. Wide excision is recommended for curative treatment to prevent recurrence and metastasis; however, this approach often results in functional impairments for patients [86,87]. In contrast, conservative excision is usually sufficient for patients with enchondromas, as these tumors rarely recur or metastasize [88]. Nevertheless, patients with low-grade chondrosarcomas may still face recurrence and metastasis after conservative excision. Thus, there is a pressing need to identify highly accurate and specific prognostic biomarkers to guide clinical management, assess tumor aggressiveness, and predict disease prognosis in these patients.

Biomarkers are essential in managing chondrosarcomas by facilitating screening, diagnosis, prognosis prediction, and monitoring of tumor progression. IDH1/2 mutations, found in approximately 39% of chondrosarcoma cases, are strongly correlated with poor survival, larger tumor size, higher grade, and increased relapse risk, positioning them as crucial prognostic indicators. Similarly, the overexpression of EphA2, a receptor implicated in angiogenesis and metastasis, has been observed in chondrosarcomas and related bone sarcomas, showing promise as a therapeutic target through its significant response to receptor inhibition [89]. Proteins involved in post-translational modification, such as SUMO2/3, are also emerging as prognostic biomarkers; their high expression levels are linked with worse overall survival, and inhibition of SUMO pathways has shown antitumor effects in preclinical studies [90]. Other markers, including esRAGE and HMGB1, correlate with tumor recurrence and poor prognosis in lower-grade chondrosarcomas. At the same time, elevated levels of Aurora kinases and hypoxia-inducible factors (HIF-1α and HIF-2α) are associated with higher-grade tumors and worse clinical outcomes. Collectively, these biomarkers enhance diagnostic precision and open new avenues for targeted therapies in chondrosarcoma management [84,91].

Several biomarkers show promise for improving chondrosarcoma diagnosis and prognosis. VEGF-A and VEGF-C, key players in angiogenesis and lymphangiogenesis, are upregulated in chondrosarcomas and may be useful for staging. AMACR helps differentiate between benign enchondromas and malignant chondrosarcomas, while periostin is present in low-grade chondrosarcomas but not in enchondromas, aiding diagnosis. miRNAs like miR-27b and miR-624–3p, which regulate VEGF-C, offer insight into tumor progression and potential therapeutic targets. However, these biomarkers need further clinical validation to confirm their effectiveness in chondrosarcoma management [92].

Additionally, microRNAs (miRNAs) play a crucial role in chondrosarcoma pathogenesis, with their dysregulation driving tumor growth and metastasis and influencing patient prognosis. Certain miRNAs, like miR-143-3p and miR-145-5p, are downregulated in chondrosarcomas, which leads to the upregulation of target genes, such as Fascin-1, thus promoting cell migration and metastasis. This relationship underscores the value of these miRNAs as biomarkers for disease progression, as their altered levels are associated with increased tumor aggressiveness. Additionally, low levels of miR-335, which usually suppresses metastasis through targets like SOX4 and Tenascin-C, correlate with poorer survival, further supporting its prognostic significance. MiRNAs, such as miR-21-5p and miR-454-3p, also influence pathways crucial for tumor proliferation and survival, including the STAT3/NF-κB axis, whose activation drives chondrosarcoma progression. By suppressing CCR7, miR-21-5p reduces STAT3 signaling, highlighting a potential therapeutic target to curb tumor growth. Restoring these downregulated miRNAs could help inhibit tumor progression and enhance the effectiveness of therapeutic interventions, making miRNAs both valuable biomarkers and potential targets for chondrosarcoma treatment [92,93].

In chondrosarcomas, FFPE-based molecular analysis shows promise in identifying novel biomarkers and therapeutic targets. FFPE analysis can help pinpoint differentially expressed genes linked to tumor progression and metastasis by comparing gene expression profiles between tumor and normal tissues. This approach may also aid in identifying chondrosarcoma-specific markers within its tumor microenvironment or pathways integral to its development. Additionally, FFPE data could facilitate meta-analyses and comparisons with other bone cancers, like osteosarcoma, offering a deeper molecular understanding of chondrosarcomas and supporting the development of targeted therapies [94].

Additionally, in a study conducted by Tudor et al., polyethylene glycol-encapsulated iron oxide nanoparticles (IONPDOX) demonstrated potential as hyperspectral biomarkers for assessing radiosensitivity in human chondrosarcoma cells. By combining IONPDOX with carbon ion or photon radiation, researchers observed enhanced cytotoxic effects, including increased DNA damage marked by micronucleus formation and distinct changes in the hyperspectral profiles of cell nuclei. These findings suggest that IONPDOX could serve as a valuable biomarker in evaluating and improving the effectiveness of radiotherapy in highly resistant tumors like chondrosarcomas [95].

## 7. Conclusions

In conclusion, identifying genetic alterations and molecular mechanisms underlying chondrosarcomas has paved the way for developing targeted therapies and prognostic biomarkers. Chondrosarcomas, largely resistant to chemotherapy and radiotherapy, present significant treatment challenges, underscoring the importance of biomarkers like VEGF, leptin, adiponectin, and periostin. These markers help distinguish between benign and malignant lesions and offer insights into tumor progression and metastasis. Although promising, these biomarkers require further clinical validation to ensure their reliability in guiding treatment decisions and improving patient outcomes. Continued research into the molecular pathogenesis of chondrosarcomas is essential for advancing personalized therapeutic strategies and enhancing prognostic accuracy.

## Figures and Tables

**Table 1:** Overview of the mechanisms and potential therapeutic implications of DNA methylation and miRNAs and their roles in chondrosarcoma progression.

Target	Mechanism	Impact	Potential Therapeutic Implications
DNA Methylation	Addition of a methyl group to DNA by DNMTs	Regulates gene expression and contributes to tumor progression	Involves genes like Satellite 1 and L1
Hypomethylation	Loss of methyl groups globally or in specific genes	Promotes tumor progression, especially in repetitive DNA sequences like Satellite 1 and L1	
DNMT Inhibitors (Azacytidine, Decitabine)	Inhibit DNA methyltransferase activity; FDA-approved for AML and MDS	Decitabine (DAC) leads to hypomethylation and increased Sox-2 and MDK expression, with mixed effects on tumor behavior	Mixed effects on tumor invasiveness in chondrosarcoma (CS) models
Decitabine and CpG Site Methylation	Reduces methylation at CpG sites, influencing key epithelial markers	Linked to MET (mesenchymal-to-epithelial transition), affecting cell adhesion, apoptosis, and cell cycle	Potential therapeutic strategy targeting epithelial markers in CS
Tumor Microenvironment	Influences DNA methylation patterns and gene expression	Hypomethylation and gene expression changes observed when SRC cells transplanted into different environments	Environment plays a complex role in CS progression
Hypermethylation and IDH Mutations	Associated with IDH1/IDH2 mutations, leading to D2HG production and widespread hypermethylation	Hypermethylation linked to stem cell maintenance and differentiation of genes in CS	Potential therapeutic targets: TET enzymes, Aurora kinase, HDAC inhibitors
Hypermethylated Genes (p16INK4, E-cadherin, NAMPT)	Silencing of tumor suppressor genes via hypermethylation	Potential for targeting NAMPT/NAPRT in high-grade chondrosarcomas	
RUNX3	Tumor suppressor gene silenced through hypermethylation	Restoring RUNX3 expression inhibits tumor growth and induces apoptosis	Target for therapeutic reactivation in CS
p73	Tumor suppressor gene (part of p53 family), often hypermethylated in CS	Correlates with CS progression; potential as a prognostic marker and therapeutic target	
miR-100	miRNA that targets mTOR	It acts as a tumor suppressor but is downregulated in CS	Targeting the mTOR pathway could have therapeutic value
miR-30a	miRNA that targets oncogene SOX4	Inhibits tumor growth and proliferation in chondrosarcoma	
miR-181a	Oncogenic miRNA that promotes VEGF expression	Drives chondrosarcoma progression by enhancing angiogenic signaling	

**Table 2:** Potential treatments and outcomes in chondrosarcomas.

Therapeutic Approach	Mechanism/Target	Findings	Study/Notes
Chemotherapy	Ineffective	Generally ineffective due to slow proliferation, multidrug resistance, and poor vascularity	No standard systemic treatment for conventional chondrosarcomas
Nivolumab (±Pazopanib)	Immune checkpoint inhibition	13% partial response rate in sarcoma patients, including dedifferentiated chondrosarcomas	Retrospective study
BMS-986253	Targets IL-8 (chemokine involved in tumor progression)	High disease control rate, but no objective response	Phase 1 trial
Zaltoprofen	Activates PPARγ	Inhibits cell proliferation and induces apoptosis in chondrosarcoma cells	Efficacy demonstrated in preclinical studies
Palbociclib	CDK4 inhibitor	Inhibits cell cycle progression, tumor cell proliferation, and invasion	CDK4 is highly expressed and associated with poor prognosis in chondrosarcomas
MLN4924	NEDD8-activating enzyme inhibitor	Demonstrated antitumor effects in cell lines and xenograft mouse models	Preclinical study
Resveratrol	Inhibits STAT3 signaling pathway	Reduced cell viability and suppressed tumor progression in chondrosarcoma cells	Preclinical study
Rapamycin/Everolimus	mTOR inhibitors	Reduced cell viability and tumor progression in both cell lines and animal models	Preclinical studies
Immunotherapy (Pembrolizumab, Nivolumab)	Targets immune checkpoint proteins (PD-1, PD-L1)	Early studies show low response rates but significant tumor regression in some high-grade cases	Promising for aggressive/metastatic chondrosarcomas resistant to conventional therapies
